# Supplementary material for: Contrasting signatures of genomic divergence during sympatric speciation
Source: Nature. 2020 Oct 28;588(7836):106–11. doi: 10.1038/s41586-020-2845-0 (PMC7759464; doi:10.1038/s41586-020-2845-0)
Supplement: Supplementary file 1 — This file contains Supplementary Notes on the inferred demographic history of the Midas cichlid species complex, the pedigree-based association and fine-mapping of the gold locus, sampling, frequencies, and population structure of gold morphs as well as mate choice experiments. [file 41586_2020_2845_MOESM1_ESM.pdf]

nature research

https://doi.org/10.1038/s41586-020-2845-0

Supplementary information

Contrasting signatures of genomic divergence during sympatric speciation

In the format provided by the authors and unedited

Supplementary notes

Contrasting signatures of genomic divergence during sympatric speciation

Andreas F. Kautt, Claudius F. Kratochwil, Alexander Nater, Gonzalo Machado-Schiaffino, Melisa Olave, Frederico Henning, Julián Torres-Dowdall, Andreas Härer, C. Darrin Hulsey, Paolo Franchini, Martin Pippel, Eugene W. Myers, Axel Meyer

Table of Contents

1. Demographic history of the Midas cichlid species complex ……………….……………2
2. Pedigree-based association and fine-mapping of the gold locus…………………..……..4
3. Sampling, frequencies, and population structure of gold morphs…………………..…..4
4. Mate choice experiments…………………………………………………………..…..5

1. Demographic history of the Midas cichlid species complex

As a first step, we investigated the demographic history of Midas cichlid species with *MSMC2*[1] using two complementary strategies. First, we reconstructed N_e trajectories through time for each species and for each lake separately (Extended Data Fig. 2). In GL Nicaragua, we found strikingly different trajectories for *A. citrinellus* and *A. labiatus* (Extended Data Fig. 2a). While the *A. citrinellus* showed relatively stable population size dynamics, *A. labiatus* experienced a strong decline in N_e around 20 kya. Recent N_e estimates (<5 kya) indicated a strong population expansion for both species, but a precise quantification of N_e at timepoints younger than 1,000 years is not possible with the method and our data at hand. In GL Managua, demographic histories showed a pronounced population decline for both *A. citrinellus* and *A. labiatus* in the timeframe between 20 and 5 kya, with a recent recovery similar to GL Nicaragua (Extended Data Fig. 2b). For all CL populations, we found a consistent and strong signal of a population bottleneck associated with the colonization of the lakes, followed by a rapid population expansion (Extended Data Fig. 2c–d). Interestingly, CL Masaya showed a much weaker founder effect than the other CL populations but appeared to have experienced a strong bottleneck after initial colonization around 1 kya (Extended Data Fig. 2c).

Second, we assessed divergence times and gene flow between pairs of species/populations. Interestingly, we found a very recent divergence between the morphologically well-distinguishable GL species *A. citrinellus* and *A. labiatus,* indicating a split time of approximately 10 kya (Extended Data Fig. 2g). When comparing the GL and CL populations, we were able to recover the temporal sequence of colonization events with good precision. CL Apoyo clearly showed the oldest divergence among CL species to their respective source population (GL Nicaragua or GL Managua), followed by CLs Xiloá, Masaya, and Apoyeque (Extended Data Fig. 2g–l). None of the CL populations showed any sign of secondary gene flow from the source lakes into the CLs. Within CLs Apoyo and Xiloá, the sequence of divergence of sympatric species was hard to disentangle and species appeared to have diversified concurrently in both CLs rapidly after the initial colonization. The only exception was the *A. sagittae* and *A. xiloaensis* pair in CL Xiloá, which showed a later divergence compared to the other species pairs (Extended Data Fig. 2k).

As described in the Methods section on *Demographic inference*, building up on the MSMC results, we investigated the demographic histories of the two species in both great lakes and the crater lake colonizations in more detail by explicitly comparing our empirical data to model-based simulations using *Fastsimcoal*[2]. According to our most supported model of great lake species divergence, the first divergence event in the Midas cichlid species complex occurred in GL Nicaragua with the split of *A. labiatus* from *A. citrinellus* ~16,700 years ago (Extended Data Table 1; assuming a generation time of 1.5 years). This split was preceded by a bottleneck in the ancestral population that shrank from ~72,400 to ~13,400 individuals. The two species then independently migrated to GL Managua ~5,000 (*A. citrinellus*) and ~5,700 (*A. labiatus*) years ago. Given the close proximity of the two lakes and the fact that they are (at least nowadays) intermittently connected by River Tipitapa, it may seem surprising that the large Midas cichlid populations in GL Managua should have been established so recently. However, Nicaragua is one of most volcanically active countries in the world – clearly

evidenced by the chain of young crater lakes that are home to the Midas cichlid species complex. In fact, geological records suggest that a volcano erupted under water within GL Managua resulting in a tsunami that swept through the lake only 3,000 – 6,000 years ago[3]. Such an event could have exterminated the prevailing fauna (at least Midas cichlids) in the lake, which may have then been re-established by migration from GL Nicaragua, as suggested by our results. This documented volcanogenic tsunami was presumably not an isolated incidence[4] and the fauna in Nicaraguan lakes has likely experienced several major catastrophic events, which could also explain the bottleneck in the ancestral population that we inferred based on our genomic data. After divergence, both species in both great lakes grew exponentially to current population sizes of ~198,200 and ~63,900 individuals of *A. citrinellus* and *A. labiatus* in GL Nicaragua, and ~497,700 and ~201,000 in GL Managua, respectively. In contrast to our *MSMC* results, which are presumably less sensitive, our *Fastsimcoal* analyses provide evidence for recent gene flow between the two species with 2.15×10^{-4} alleles per generation in GL Nicaragua and 1.63×10^{-4} alleles per generation in GL Managua (symmetric migration rates). This rate of gene flow is up to one order of magnitude higher than gene flow between the GL populations of either species (2.5×10^{-5} between *A. labiatus* from GL Nicaragua and GL Managua and 8.3×10^{-5} between the two *A. citrinellus* GL populations). We note that migration rates are averaged over time, including times when the population sizes were much smaller than the current ones. Thus, we caution that they do not readily reflect (i.e. they overestimate) current migration rates.

In formulating models of crater lake colonizations, we used our ancestry proportion inference results (Extended Data Fig. 1l-z) to determine the most likely source populations of each crater lake. Consequently, and in concordance with their physical proximity to the great lakes, we modeled the colonization of CL Apoyo to have occurred from GL Nicaragua, the one of CL Masaya from both great lakes, and for all other CLs, Xiloá, Apoyeque, As. Managua, As. León, and Tiscapa, from GL Managua (Extended Data Table 1, Extended Data Fig. 3). In all cases did we allow for a mixed colonization by both source lake species, *A. citrinellus* and *A. labiatus* – except for CL Masaya in which case the model was already very complex due to the colonization by both great lakes – and indeed, we consistently found a smaller, but non-zero, contribution from *A. labiatus* (Extended Data Table1, Extended Data Fig. 3) – the rarer of the two species in the great lakes. According to our results, CL Apoyo was the first of the CLs to be colonized by Midas cichlids ~4,700 years ago, followed by CL Xiloá ~4,300, CL Masaya ~2,800, As. León ~1,700, As. Managua ~1,200, CL Apoyeque ~900, and CL Tiscapa ~800 years ago (Extended Data Table 1, Extended Data Fig. 3). We note that these inferred colonization times do not necessarily represent the first ever colonization times, but those of populations that have persisted to the present day (earlier populations may have gone extinct due to catastrophic events as argued above). Given the already high model complexities and due to limitations in representing the data of several populations in multidimensional site frequency spectra, we included only two species and therefore one intra-lacustrine divergence event in the two multispecies CLs Apoyo (*A. zaliosus* vs. *A. astorquii*) and Xiloá (*A. amarillo* and *A. sagittae*). The intralacustrine divergence event included the first species to split in each CL (based on Extended Data Fig. 1a). In the case of CL Apoyo, this sympatric speciation event occurred ~3,700 years ago, in the case of CL Xiloá ~2,700 years ago. We detected evidence for low (an order of magnitude lower than between the great lake species), but non-zero, gene flow between the sympatric species in both CLs (8.7×10^{-5} in CL Apoyo and 5.8×10^{-5} in CL Xiloá), in agreement with our mate choice experiments of these species that showed high, but not complete, assortative mating in the laboratory. Also consistent with previous results based on RADseq data[5], we found strong support for secondary waves of colonization from the respective source great lakes prior to these intralacustrine divergence events (Extended Data Table 1). Interestingly, similar findings have recently been reported in Lord Howe palm trees[6]

and earlier in cichlid fish in Crater Lake Massoko[7]. It is important to note that the fact that some gene flow / admixture may have occurred is not sufficient to reject a case of sympatric speciation[8].

Along these lines, we also note that we found that the great lake species ancestry is shared equally among all sympatric species in CLs Apoyo and Xiloá (Extended Data Table 1, Extended Data Fig. 3), as would be expected during sympatric speciation compared to unequal ancestry proportions among sympatric species that might indicate that pre-existing reproductive barriers might have facilitated speciation. Thus, we find no evidence that the colonization by both great lakes species and subsequent mixing/hybridization or secondary waves of colonization were directly responsible for diversification within CLs Apoyo and Xiloá[8]. The differential sorting of the two great lake species would in any case not be sufficient to explain the origin of more than two species (i.e. six and four species in CLs Apoyo and Xiloá, respectively). Instead, altogether, our results are consistent with sympatric speciation in CLs Apoyo and Xiloá. Finally, and importantly, we also found such admixture signals and evidence for a colonization by both species for all other crater lakes, irrespective of the presence of multiple species, similar to what we found before based on RADseq data[9].

Our results are overall fully compatible with the geological history of the lakes. The estimated colonization time (Fig. 1a, Extended Data Table 1, Extended Data Fig. 3) of a crater lake is in each case close to or younger than its maximum age (summarized in [10]). Moreover, as reported above, the estimated colonization of CL Managua coincides with major eruptions that happened 3,000–6,000 years ago[3] and likely represents a re-colonization event of the lake.

2. Pedigree-based association and fine-mapping of the gold locus

A total of 41 normal and 124 gold F_2s were obtained from a cross of a golden (G) CL Masaya individual and a dark (D) GL Nicaragua individual. This proportion is in concordance with a ratio of 1:3 ($\chi_2 = 0.049$, df = 1, $P = 0.823$), expected for a dominant Mendelian trait. All F_1 and F_2 individuals were normally pigmented for several months post-hatching. All F_1s and most of the F_2s underwent a transition phase of melanophore cell death as previously reported[11,12] until the entire body and fins become orange or even whitish (gold morph, "G") (Fig. 1d). Interestingly, some individuals transitioned as soon as 6-12 months, while most individuals took over 1-2 years. To assess if the transition time differs between DG and GG individuals, we set up a second cross between two heterozygote F2s. At 12 months of age, the ratio between golden and dark fish was very close to 1:1 (36:34) and significantly different from 2:1 ($\chi_2 = 7.314$, df = 1, $P < 0.01$) at 12 months of age. At 20 months of age, the proportion of heterozygotes and homozygotes (80:34) was no longer significantly different from the expected Mendelian proportions of 2:1 ($\chi_2 = 0.63$, df = 1, $P = 0.43$). This implies that there is a dosage effect and that *GG* homozygotes undergo morphological color change earlier than *GD* heterozygotes. Such a dosage dependent variation in onset could impede potential predation pressures[13,14] but also influence assortative mating[15] (if color is the mating cue) and therefore divergence.

3. Sampling, frequencies, and population structure of gold morphs

The frequencies of gold morphs in lakes can vary between likely 0% (CL Tiscapa) or effectively <<1% (CL Apoyo, only one golden individual was ever observed during field trips; n of observations > 1000 and CL As. León: three individuals observed) up to 5-20% (other lakes). Interestingly, we found substantial genomic differentiation (genome-wide F_{ST}: 0.071; Table S10) between gold and dark morphs in CL As. León, yet with the reported very low frequency of golden individuals (Extended Data Fig. 4o) and only three golden individuals in our dataset (the only ones ever caught in this CL) this result has to be interpreted with caution and was therefore not emphasized in the main text.

4. Mate choice experiments

Sympatric speciation is thought to be strongly facilitated if traits under divergent selection lead automatically to pre-mating isolation[16,17]. In their simplest form, these so-called magic traits lead to the spatial or temporal isolation of individuals that are adapted to different habitats. In another (non-exclusive) form, pre-mating isolation can be facilitated if the traits under selection act simultaneously as cues for mate choice. While theoretically appealing, the role of magic traits in (sympatric) speciation is currently unclear due to a paucity of empirical studies.

Midas cichlids are a very interesting system to address this question as they have diversified along multiple phenotypic axes, some of which are thought to play a significant role in mate choice[15,18]. Midas cichlids in Nicaragua form seasonally monogamous pairs that breed at the shore in caves, sand, algae, or under large rocks, and both parents protect the brood for an extensive time period that might last more than a month[19]. In particular, much has been debated about the role of body color (dark/gold), lip size, and body shape as cues for mating in Midas cichlids.

Midas cichlids do mate assortatively with regard to the dark/gold coloration polymorphism (Extended Data Table 2). For example, in CL Xiloá, pairs in which both individuals are golden are more common (4% and 8% for *A. sagittae* and *A. xiloaensis*, respectively) than predicted given the low frequency of golden fish (0.5% and 4%, respectively[15]). This result appears to be consistent in the wild and in the lab, and across different species[19,20]. Yet, there is still a large number of disassortative couples that likely lead to gene flow and thereby decrease or prevent overall differentiation between the color morphs. For example, in CL Xiloá disassortative couples represent over 5% and 23% of all couples of *A. sagittae* and *A. xiloaensis* that we observed in that CL, respectively[15].

Similar patterns were observed when exploring if pair formation happens according to lip size (Extended Data Table 2). A field survey in CL Apoyeque found strong departures from random mating between fish with thin and thick lips[18]. The number of assortative couples found was significantly higher than expected given the relative abundance of both morphs and assuming random mating (Extended Data Table 2). When this experiment was repeated under laboratory conditions and using Midas cichlids from the great lakes, not a single disassortative couple was formed[18]. A similar pattern was previously reported from laboratory experiments determining mate choice in small groups of fish consisting of a mix of *A. labiatus* from GL Nicaragua and *A. citrinellus* from CL Masaya[21]. Six out seven pairs mated assortatively in this case (Extended Data Table 2).

Less effort has been put into determining the role of body morphology on Midas cichlids mate choice. Being shallow and turbid, the great lakes represent a rather homogeneous habitat, which differs markedly from the deep clear waters of the CLs[10,20]. In the CLs, the water basins are more clearly divided into substrate-associated (benthic) and open-water (limnetic) zones[22,23]. Stemming from a more generalist great lake ancestor, the limnetic niche has apparently been filled within the last few thousand years in CL Apoyo by *A. zaliosus* and in CL Xiloá by *A. sagittae*. These limnetic species differ in several ecologically important traits from the other species in their respective CL and demonstrate a parallel pattern of divergent body shape evolution that happened independently in both the CL Apoyo and CL Xiloá radiation[24]. Field surveys have not found disassortative pairs among species in either CL Apoyo[25] or CL Xiloá[15]. These findings are consistent with the prediction that pair formation might occur in the species' respective feeding habitats, as pre-mating isolation via habitat isolation was a necessary condition of a theoretical model tailored to the radiation of Midas cichlids in CL Apoyo[26]. This assumption had, however, not been tested before.

In order to test the hypothesis of pre-mating isolation due to divergence in habitat preference we performed intra-lacustrine mate choice experiments for two different crater

lakes: Apoyo and Xiloá. The experimental arena used in this experiment consisted of a 340 x 160 x 80 cm (length x width x depth) volume within a larger tank (595 x 240 x 80 cm). This experimental arena was further divided into five compartments: a large one (340 x 80 x 80 cm) and four small ones (85 x 80 x 80 cm). The four small compartments were connected to the larger one but separated from each other by a divider. Thus, the small compartments had three sides closed and one opened to the large compartment. The center of each small compartment contained a large flowering pot that served as spawning site and therefore constituted a defendable territory. The large compartment was open and had no defendable areas; however, fish were free to breed anywhere in the experimental tank and indeed in a few occasions they did breed in the large compartment by creating spawning sites in the corners by removing gravel from the ground. All walls of the experimental arena were white, and the bottom was covered by 5 to 10 cm of coarse gravel (i.e., pebbles of 20 to 60 mm size) of a greyish coloration. Water temperature was maintained at 28 ± 1°C. The experimental arena was illuminated with artificial fluorescent light in a 12:12 h on-off daily cycle.

When given the opportunity to mate with a conspecific, we found that individuals of the two species *A. zaliosus* and *A. astorquii* from CL Apoyo mated almost exclusively assortatively: only two out of 19 pairs were of a mixed constellation, significantly deviating from a pattern of random mating ($\chi2 = 11.842$, $P = 0.00058$). Of the assortative pairs, ten were *A. zaliosus* and seven *A. astorquii*. The two disassortative pairs included both possible combinations of sex and species. Between species from CL Xiloá, we did not detect a single disassortative pair in the intra-lacustrine experiment. In total, eight assortative pairs of *A. amarillo* and three of *A. sagittae* formed in this experiment before it had to be terminated due to a shortage of replacements for females of *A. amarillo*. While the sample size was relatively low for this experiment, the pair composition significantly deviated from random expectations ($\chi2$ goodness of fit test, $\chi2 = 12$, $P = 0.00053$). Thus, even in the absence of different habitats the two sympatric species pairs from both CLs mate assortatively. This suggests that divergent mate preferences act as a strong reproductive pre-mating barrier in these species. Habitat isolation, on the other hand, is consequently not a necessary barrier for assortative mating in the sympatric CL Midas cichlid species of CLs Apoyo and Xiloá.

Since i) mating was assortative between limnetic and benthic species form the same lake, but ii) habitat isolation does not constitute a necessary condition for pre-mating isolation (at least not anymore), and iii) the species pairs have diverged along parallel phenotypic trajectories in both lakes[24], we wanted to further investigate whether mate preference would also follow a parallel pattern. In other words, we wanted to test whether fish would mate assortative by ecomorph even when exposed to fish from a different lake[27,28]. To test this hypothesis of "parallel speciation" (sensu[29]), we conducted two inter-lacustrine experiments. In these phylogenetically-controlled experiments – fish in both lakes form monophyletic radiations and every single CL Apoyo species is equally distantly related to any of the CL Xiloá species, independent of ecomorph – significant positive assortative mating would provide evidence that traits that have diverged independently in both lake radiations do also constitute cues for mate choice. If these traits play no role in mate choice, we would expect mating to be random with respect to ecomorph. In the first trial (i.e. with females from CL Xiloá and males from CL Apoyo) and counting again only fish that could still be replaced by naïve individuals, we detected seven assortative (limnetic x limnetic, or benthic x benthic) and five disassortative (limnetic x benthic) pairs. This pattern was indistinguishable from random expectations ($\chi2$ goodness of fit test, $\chi2 = 0.333$, $P = 0.5637$) and was not explained by a single species: seven pairs involved females of *A. sagittae* and five females of *A. amarillo*. The experiment had to be terminated due to a shortage of *A. zaliosus* males for replacement at this point. Yet, to obtain a larger sample size we removed all remaining *A. zaliosus* males in the tank and continued the experiment until the next group of replacements (*A. amarillo* females) was depleted. Three

more assortative pairs (*A. amarillo* female x *A. astorquii* male) and two more disassortative pairs (*A. sagittae* female x *A. astorquii* male) were obtained, again following a random pattern.

In the second trial (i.e. with females from CL Apoyo and males from CL Xiloá), we found that fish mated disassortatively with respect to ecomorph (χ_2 = 18.75, P = 0.000015). Most pairs (17 out of 27) were composed of a female of the limnetic species from CL Apoyo (*A. zaliosus*) and a male of the benthic species from CL Xiloá (*A. amarillo*). Eight pairs were disassortative in the opposite direction (females of the benthic species of CL Apoyo and males of the limnetic species from CL Xiloá). At the time of the experiment, *A. zaliosus* females were slightly larger than those of *A. astorquii* (mean *A. zaliosus* = 152.6 ± 29.6 g.; mean *A. astorquii* = 130.0 ± 19.9 g.; t-test = 2.054, P = 0.0506). To determine if *A. astorquii* females preferred to mate disassoratively by ecomorph, or it was a consequence of *A. zaliosus* females' choice, we removed all remaining *A. zaliosus* females in the tank and continued the experiment. Twelve more pairs were formed, seven were assortative by ecomorph, whereas five were disassortative. This pattern was indistinguishable from random mating (χ_2 = 0.333, P = 0.5637). Thus, the results of our two inter-lacustrine experiments strongly suggest that while the traits under divergent selection – such as body shape – have evolved in parallel in both CLs, the traits responsible for mate discrimination have not.

Supplementary references

1 Malaspinas, A. S. *et al.* A genomic history of Aboriginal Australia. *Nature* **538**, 207-214, doi:10.1038/nature18299 (2016).

2 Excoffier, L., Dupanloup, I., Huerta-Sanchez, E., Sousa, V. C. & Foll, M. Robust demographic inference from genomic and SNP data. *Plos Genet* **9**, e1003905, doi:10.1371/journal.pgen.1003905 (2013).

3 Freundt, A., Kutterolf, S., Wehrmann, H., Schmincke, H. U. & Strauch, W. Eruption of the dacite to andesite zoned Mateare Tephra, and associated tsunamis in Lake Managua, Nicaragua. *J Volcanol Geoth Res* **149**, 103-123, doi:10.1016/j.jvolgeores.2005.06.001 (2006).

4 Freundt, A., Strauch, W., Kutterolf, S. & Schmincke, H. U. Volcanogenic tsunamis in lakes: Examples from Nicaragua and general implications. *Pure Appl Geophys* **164**, 527-545, doi:10.1007/s00024-006-0178-z (2007).

5 Kautt, A. F., Machado-Schiaffino, G. & Meyer, A. Multispecies outcomes of sympatric speciation after admixture with the source population in two radiations of Nicaraguan crater lake cichlids. *Plos Genet* **12**, e1006157, doi:10.1371/journal.pgen.1006157 (2016).

6 Osborne, O. G. *et al.* Speciation in Howea Palms Occurred in Sympatry, Was Preceded by Ancestral Admixture, and Was Associated with Edaphic and Phenological Adaptation. *Mol Biol Evol* **36**, 2682-2697, doi:10.1093/molbev/msz166 (2019).

7 Malinsky, M. *et al.* Genomic islands of speciation separate cichlid ecomorphs in an East African crater lake. *Science* **350**, 1493-1498, doi:10.1126/science.aac9927 (2015).

8 Richards, E. J., Servedio, M. R. & Martin, C. H. Searching for Sympatric Speciation in the Genomic Era. *Bioessays* **41**, e1900047, doi:10.1002/bies.201900047 (2019).

9 Kautt, A. F., Machado-Schiaffino, G. & Meyer, A. Lessons from a natural experiment: Allopatric morphological divergence and sympatric diversification in the Midas cichlid species complex are largely influenced by ecology in a deterministic way. *Evol Lett* **2**, 323-340, doi:10.1002/evl3.64 (2018).

10 Elmer, K. R., Kusche, H., Lehtonen, T. K. & Meyer, A. Local variation and parallel evolution: morphological and genetic diversity across a species complex of neotropical crater lake cichlid fishes. *Philos T R Soc B* **365**, 1763-1782, doi:10.1098/rstb.2009.0271 (2010).

11 Dickman, M. C., Schliwa, M. & Barlow, G. W. Melanophore death and disappearance produces color metamorphosis in the polychromatic Midas cichlid (Cichlasoma citrinellum). *Cell Tissue Res* **253**, 9-14, doi:10.1007/BF00221733 (1988).

12 Henning, F., Jones, J. C., Franchini, P. & Meyer, A. Transcriptomics of morphological color change in polychromatic Midas cichlids. *Bmc Genomics* **14**, 171, doi:10.1186/1471-2164-14-171 (2013).
13 Torres-Dowdall, J., Golcher-Benavides, J., Machado-Schiaffino, G. & Meyer, A. The role of rare morph advantage and conspicuousness in the stable gold-dark colour polymorphism of a crater lake Midas cichlid fish. *Journal of Animal Ecology* **86**, 1044-1053, doi:10.1111/1365-2656.12693 (2017).
14 Kusche, H. & Meyer, A. One cost of being gold: selective predation and implications for the maintenance of the Midas cichlid colour polymorphism (Perciformes: Cichlidae). *Biological Journal of the Linnean Society* **111**, 350-358, doi:10.1111/bij.12205 (2014).
15 Elmer, K. R., Lehtonen, T. K. & Meyer, A. Color Assortative Mating Contributes to Sympatric Divergence of Neotropical Cichlid Fish. *Evolution* **63**, 2750-2757, doi:10.1111/j.1558-5646.2009.00736.x (2009).
16 Maynard Smith, J. Sympatric Speciation. *Am Nat* **100**, 637-650, doi:10.1086/282457 (1966).
17 Gavrilets, S. *Fitness landscapes and the origin of species*. (Princeton University Press, 2004).
18 Machado-Schiaffino, G. *et al.* Incipient speciation driven by hypertrophied lips in Midas cichlids fish? *Mol Ecol* **26**, 2348-2362, doi:10.1111/mec.14029 (2017).
19 McKaye, K. R. & Barlow, G. W. in *Investigations of the ichthyofauna of Nicaraguan lakes* (ed Thomas B. Thorson) Ch. 34, 465-475 (University of Nebraska-Lincoln, 1976).
20 Barlow, G. W. in *Investigations of the ichthyology of Nicaraguan lakes* (ed Thomas B. Thorson) 333-358 (University of Nebraska Press, 1976).
21 Baylis, J. R. Quantitative Study of Long-Term Courtship: 1. Ethological Isolation between Sympatric Populations of the Midas Cichlid, *Cichlasoma citrinellum*, and the Arrow Cichlid, *C. zaliosum*. *Behaviour* **59**, 59-69, doi:10.1163/156853976X00460 (1976).
22 Vivas, R. & McKaye, K. R. Habitat selection, feeding ecology, and fry survivorship in the *Amphilophus citrinellus* species complex in Lake Xiloá, Nicaragua. *Journal of Aquariculture and Aquatic Sciences* **IX**, 32-48 (2001).
23 Barluenga, M., Stolting, K. N., Salzburger, W., Muschick, M. & Meyer, A. Sympatric speciation in Nicaraguan crater lake cichlid fish. *Nature* **439**, 719-723, doi:10.1038/Nature04325 (2006).
24 Elmer, K. R. *et al.* Parallel evolution of Nicaraguan crater lake cichlid fishes via non-parallel routes. *Nat Commun* **5**, doi:10.1038/Ncomms6168 (2014).
25 Stauffer, J. R., McCrary, J. K. & Black, K. E. Three new species of cichlid fishes (Teleostei: Cichlidae) from Lake Apoyo, Nicaragua. *Proceedings of the Biological Society of Washington* **121**, 117-129, doi:10.2988/06-37.1 (2008).
26 Gavrilets, S., Vose, A., Barluenga, M., Salzburger, W. & Meyer, A. Case studies and mathematical models of ecological speciation. 1. Cichlids in a crater lake. *Mol Ecol* **16**, 2893-2909, doi:10.1111/j.1365-294X.2007.03305.x (2007).
27 Rundle, H. D., Nagel, L., Boughman, J. W. & Schluter, D. Natural selection and parallel speciation in sympatric sticklebacks. *Science* **287**, 306-308, doi:10.1126/science.287.5451.306 (2000).
28 Nosil, P., Crespi, B. J. & Sandoval, C. P. Host-plant adaptation drives the parallel evolution of reproductive isolation. *Nature* **417**, 440-443, doi:10.1038/417440a (2002).
29 Schluter, D. & Nagel, L. M. Parallel Speciation by Natural Selection. *Am Nat* **146**, 292-301, doi:10.1086/285799 (1995).
