# Supplementary material for: Contrasting signatures of genomic divergence during sympatric speciation
Source: Nature. 2020 Oct 28;588(7836):106–11. doi: 10.1038/s41586-020-2845-0 (PMC7759464; doi:10.1038/s41586-020-2845-0)
Supplement: Supplementary file 2 — Reporting Summary [file 41586_2020_2845_MOESM2_ESM.pdf]

nature research

Corresponding author(s): Axel Meyer

Last updated by author(s): Jul 13, 2020

Reporting Summary

Nature Research wishes to improve the reproducibility of the work that we publish. This form provides structure for consistency and transparency in reporting. For further information on Nature Research policies, see Authors & Referees and the Editorial Policy Checklist .

Statistics

For all statistical analyses, confirm that the following items are present in the figure legend, table legend, main text, or Methods section.

n/a	Confirmed	
☐	☒	The exact sample size (*n*) for each experimental group/condition, given as a discrete number and unit of measurement
☐	☒	A statement on whether measurements were taken from distinct samples or whether the same sample was measured repeatedly
☐	☒	The statistical test(s) used AND whether they are one- or two-sided<br>*Only common tests should be described solely by name; describe more complex techniques in the Methods section.*
☒	☐	A description of all covariates tested
☒	☐	A description of any assumptions or corrections, such as tests of normality and adjustment for multiple comparisons
☐	☒	A full description of the statistical parameters including central tendency (e.g. means) or other basic estimates (e.g. regression coefficient) AND variation (e.g. standard deviation) or associated estimates of uncertainty (e.g. confidence intervals)
☐	☒	For null hypothesis testing, the test statistic (e.g. *F*, *t*, *r*) with confidence intervals, effect sizes, degrees of freedom and *P* value noted<br>*Give P values as exact values whenever suitable.*
☒	☐	For Bayesian analysis, information on the choice of priors and Markov chain Monte Carlo settings
☒	☐	For hierarchical and complex designs, identification of the appropriate level for tests and full reporting of outcomes
☒	☐	Estimates of effect sizes (e.g. Cohen's *d*, Pearson's *r*), indicating how they were calculated

Our web collection on statistics for biologists contains articles on many of the points above.

Software and code

Policy information about availability of computer code

Data collection

Geometric morphometric landmark data acquisition was performed with tpsDig v.2.32. Lip size and maximum pharyngeal jaw tooth size were measured from photographs with Fiji (ImageJ) v.2.0.0.

Data analysis

Genome assembly and annotation:
MARVEL and custom code for genome assembly (https://github.com/MartinPippel/DAmar), DBdust (https://github.com/thegenemyers/DAZZ_DB; commit: 0bd5e07), datander and TANmask (MARVEL developmental branch), daligner (https://github.com/thegenemyers/DALIGNER), Bionano Solve v3.1, pbalign (https://github.com/PacificBiosciences/pbalign; commit: 0669a4e;), blasr (5.3.2-a579bd5), 3d-dna (https://github.com/theaidenlab/3d-dna; commit 5baf854), Juicer v.1.7.6, Arrow (https://github.com/PacificBiosciences/GenomicConsensus; commit c92ef5d), freebayes v.1.1.0, samtools v.1.8, bcftools v.1.7 consensus, LASTZ v.1.02.00, EvidenceModeler v.1.1.1, Braker v.2.0.4, HISAT v.2.1.0, Stringtie v.1.3.3b, exonerate v.2.4.0, PASA v.2.0.2, Trinity v.2.6.0, Cufflinks v.2.2.1, BLASTp v.2.2.31, gVolante v.1.2.1, BUSCO v.2/v3, gffreads v.0.11.4

Population genomics, phylogenomics, GWAS:
Picard tools v.2.9.4, BWA mem v.0.7.15, freebayes v.1.1.0, vcftools v.0.1.1, plink v.1.90/v. 2.00, gem-mappability v.1.315 (GEM library), SHAPEIT2 v.2.r900, FastEPRR v.2.0, RAxML v.8, ASTRAL III v5.6.1, BEAST v.2.4.7, PhyParts v.0.0.1, ChromoPainter v.2, GLOBETROTTER v.1, MSMC v.2.1.2, Fastsimcoal v.2.6, ANGSD v.0.929, EIGENSOFT v.7.2.1, Admixture v.1.3.0, EMMAX beta-07Mar2010, Saguaro r44, pegas v.0.11 (R package), Relate v.1.0.16, MSMS v.1.3, libsequence v.1.9.8, BEDTools v2.29.2, ShinyGO v.0.61, REHH v.2.0.2 (R package), dadi v.1.7.0 (python package), custom code (https://github.com/alexnater/midas-genomics)

QTL mapping:
Trimmomatic v.0.36, PicardTools v.1.141, bwa-mem v.0.7.15, freebayes v.1.3.0, JoinMap v.4.0, R/qtl (R package)

Geometric morphometrics and partial least squares regressions:
geomorph v3.0.6 (R package), MASS v.7.3 (R package), plsdepot v.0.1.17 (R package), custom code (https://github.com/alexnater/midas-

genomics)

For manuscripts utilizing custom algorithms or software that are central to the research but not yet described in published literature, software must be made available to editors/reviewers. We strongly encourage code deposition in a community repository (e.g. GitHub). See the Nature Research guidelines for submitting code & software for further information.

Data

Policy information about availability of data

All manuscripts must include a data availability statement. This statement should provide the following information, where applicable:

- Accession codes, unique identifiers, or web links for publicly available datasets
- A list of figures that have associated raw data
- A description of any restrictions on data availability

The genome assembly has been deposited at DDBJ/ENA/GenBank under accession JACBYM000000000. The version described in this paper is version JACBYM010000000. Whole-genome resequencing data of all 453 samples in form of unmapped BAM files (PRJEB38173) and previously unpublished transcriptomic data (PRJNA635556) have been deposited to ENA and NCBI/SRA, respectively. Geometric morphometric data, information on samples, and downstream data to reproduce our results can be downloaded from Dryad (10.5061/dryad.bcc2fqz91).

Field-specific reporting

Please select the one below that is the best fit for your research. If you are not sure, read the appropriate sections before making your selection.

☐ Life sciences ☐ Behavioural & social sciences ☒ Ecological, evolutionary & environmental sciences

For a reference copy of the document with all sections, see nature.com/documents/nr-reporting-summary-flat.pdf

Ecological, evolutionary & environmental sciences study design

All studies must disclose on these points even when the disclosure is negative.

Study description	This study comprises a de novo genome assembly (single sample), whole-genome re-sequencing of 453 samples (sample sizes of 10-24 per species/lake), analysis of a mapping panel for determining the genetic basis of the dark/gold polymorphism (one cross), a quantitative trait locus mapping panel for pharyngeal jaws and body morphology (one cross), and mate choice experiments (two experiments with two trials each. For the mate choice experiments, the first experiment tested mate choice between sympatric species from two different lakes (with trials corresponding to the two lakes). The second experiment tested mate choice between allopatric species (trials one and two corresponded to fish from one sex of lake A vs. fish of the other sex from lake B and vice versa). Each trial was analyzed independently and the response variable was categorical with the two levels assortative or disassortative. The analysis tested the probability of assortative over disassortative mating.
Research sample	This work is based on a new, high-quality reference genome of Amphilophus citrinellus and 453 re-sequenced genomes that were sampled between 2003 and 2015. We aimed to sample at least 20 individuals per species, lake and/or ecomorph whenever possible. Sample sizes for the resequenced genomes (in brackets) are: GL Nicaragua A. citrinellus [24], GL Nicaragua A. labiatus [24], GL Managua A. citrinellus [25], GL Managua A. labiatus [25], CL Apoyeque A. cf. citrinellus (thin-lipped and thick-lipped) [20+20], CL Apoyo A. astorquii [23], CL Apoyo A. chancho [16], CL Apoyo A. flaveolus [16], CL Apoyo A. globosus [25], CL Apoyo A. supercilius [10], CL Apoyo A. zaliosus [21], CL Apoyo admixed individuals [9], CL As. León A. cf. citrinellus [20], CL As. Managua A. tolteca [20], CL Xiloá A. amarillo [21], CL Xiloá A. sagittae [27], CL Xiloá A. viridis [24], CL Xiloá A. xiloaensis [16], CL Xiloá admixed individuals [14], CL Masaya A. cf. citrinellus (thin-lipped and thick-lipped) [20+20], CL Tiscapa A. cf. citrinellus [20]. Age could not be determined reliably in wild-caught fish, but all sampled individuals were, based on their size, adult fish (standard length =14.54 ± 3.43 cm (mean ± SD)). Whenever possible, we determined the sex of individuals. Of those that could be sexed, 58% were males and 42% females.<br>We used existing RNA-seq data sets from lip tissue (Manousaki et al. 2013; doi: 10.1111/mec.12034), and whole-body extractions at 1-day (Franchini et al. 2019; doi:10.1093/molbev/msz168) and 1-month post-hatch (Franchini et al. 2016; doi:10.1093/gbe/evw097) for the generation of the genome annotation.<br>For mate choice experiments, we used a single brood of wild-caught fish for each of the four studies species. All fish of these broods that survived to adulthood were considered in this study. Individuals in each trial were selected randomly.
Sampling strategy	Fish were caught with gill nets or by harpooning in crater lakes Asososca León, Asososca Managua, Apoyeque, Apoyo, Masaya Tiscapa, and Xiloá. Fish or tissue samples from the great lakes Nicaragua and Managua were obtained from local fishermen, mostly the big fish market in Granada (Lake Nicaragua fish) and Mateares (Lake Managua fish). To obtain sufficient power (estimated based on previous studies in non-model organisms; the necessary sample size is impossible to assess in advance as it depends, for example, on the genetic architecture of the respective trait or on demographic parameters that were unknown prior to this study) for the conducted analyses we aimed to sample around 20 individuals per species, lake and/or ecomorph. For dark/gold and thin-/thick-lipped morphs we also aimed for balanced sample numbers whenever possible. However, several species and morphs are very rare in certain lakes, so sample size was dictated by the number of available samples. In these cases we aimed to include the maximum number of available samples.
Data collection	Adult fish were collected in field expeditions of the Meyer lab to Nicaragua between 2003 and 2015. Photographs were taken in the field using a digital camera. Tissues (fin and muscle tissue) were dissected with scissors and scalpels and stored in pure Ethanol before DNA extraction. The following authors participated in field trips: Axel Meyer, Andreas F. Kautt, Andreas Härer, Gonzalo Machado-Schiaffino, and Julian Torres-Dowdall. Genomic libraries preparation and data generation was performed at the University of Konstanz, the Max Planck Institute of Molecular Cell Biology and Genetics in Dresden, BGI Hong Kong, Rockefeller University, and PhaseGenomics.

For mate choice experiment, data was collected by trapping the couple defending a nest (most commonly with eggs) and determining the identity of the fish by reading their individual transponders (all adult fish at the University of Konstanz Animal Facility are individually tagged with a transponder). After pair formation, fish were removed from the experiment and relocated into stock tanks. These experiments were performed by Andreas F. Kautt, Gonzalo Machado-Schiaffino, and Julian Torres-Dowdall.

Timing and spatial scale

Adult fish were collected in field expeditions of the Meyer lab to Nicaragua between 2003 and 2015. To maximally reduce allometric effects as well as to avoid sampling untransformed golden Midas cichlid fish (that may transform only after >1 year), only adult fish were sampled. We focused on Nicaraguan lakes that were known to harbor natural populations of Midas cichlids based on previous studies. Excursions with sampling have been conducted in several years between 2003 and 2015, as sampling included many lakes and certain species and morphs were difficult to obtain in sufficient numbers (see sampling strategy).

Data exclusions

Whenever data were excluded (e.g. lakes, traits, SNPs, genomic regions) this is indicated and described in the text. Specifically, lakes were excluded when there was no variation in focal traits (e.g. golden-colored fish). For the partial least squares regression, dark/gold coloration was also excluded for lakes where golden fish are very rare (<1%; CL Apoyo and CL As. Leon). Regarding phenotypic traits — as discussed in the main text — we focused on four major axes of divergence (body shape, pharyngeal jaws, lip size, and dark/gold coloration). The criteria used for variant calling and filtering were based on established bioinformatic pipelines, that is, we applied standard quality filters (mapping quality >=30, base quality >=20). Hard variant site filters were applied using the vcffilter script from the vcflib package (https://github.com/vcflib/vcflib) (-s -f "QUAL > 1 & QUAL / AO > 10 & SAF > 0 & SAR > 0 & RPR > 1 & RPL > 1") to remove low-quality variant sites. Unplaced scaffolds showed signs of low quality including aberrantly high SNP density and heterozygosity and were therefore excluded from analyses. We hard-masked the following sites in the assembly: i) sites with a sequencing coverage across all Midas cichlid samples more than four standard deviation above the mean; ii) sites with a mappability score of less than 0.5. Mappability was calculated with the gem-mappability program v.1.315 of the GEM library74, using a k-mer size of 150 bp and allowing for up to two mismatches; iii) sites within 5 bp of an InDel variant; iv) sites within annotations of repetitive regions (repCov2), gaps, low complexity regions, or tandem repeats produced by MARVEL; v) sites in non-overlapping 10-kb windows with an average root mean square of mapping quality less than 30. In total, we masked 37.99% of all sites in the reference genome. For the machine learning analyses to detect divergent selection, we excluded regions that were not present (due to filtering) in all comparisons to allow direct comparison among species and lake populations.

Reproducibility

To test and demonstrate reproducibility and robustness we used bootstrap replication for:
a) species tree inference (gene trees were obtained with RaxML v.8 using the rapid bootstrap analysis and search of best-scoring maximum likelihood tree (option a) under a GTR+G substitution model and including 100 bootstrap replicates. Subsequent species tree estimation was inferred using ASTRAL III v5.6.1, from all individual unrooted gene trees under the multi-species coalescent model. A total of 200 bootstrap trees were obtained and used to plot the density tree.)
b) demographic inference (to estimate confidence intervals around the maximum likelihood parameter point estimates, we applied a parametric bootstrapping approach.)
c) partial least squares regressions (1,000 non-parametric bootstrap replicates (i.e. resampling with replacement))

For the genome-scale coalescent simulations we conducted 100 simulations of entire genomes (both with estimated and modified parameters), which were very consistent and showed a close fit to those in our empirical data.

We performed two mate choice (sub)experiments (intra-lacustrine and inter-lacustrine mate choice) that consisted of two trials each. In the first trial of the first experiment (intra-lacustrine mate choice), 114 adult fish (>2 years old) were available. A total of 48 of them were tested, comprising the limnetic species A. zaliosus (nfemales = 25 available (13 tested), nmales = 13 (12)) and the benthic species A. astorquii (nfemales = 30 (10), nmales = 46 (13)) from CL Apoyo. In the second trial, 115 (33 tested) adult fish were available, comprising the limnetic species A. sagittae (nfemales = 26 (6), nmales = 17 (6)) and the benthic species A. amarillo (nfemales = 12 (11), nmales = 60 (10)) from CL Xiloá. For these experiment we were restricted to using a single brood from wild-caught parents per species. In a second experiment, we aimed to test whether fish would mate assortatively by ecomorph even when exposed to fish from a different lake (inter-lacustrine mate choice). To test this hypothesis, we exposed five females of each of the limnetic and benthic species from one of the two crater lakes CL. Apoyo and Xiloá (the only two lakes harboring small adaptive radiations) to five males of each of the limnetic and benthic species from the other lake. Again, this was conducted in two different trials, one with females from CL Xiloá (nA. sagittae = 26 available (12 finally tested), nA. amarillo = 12 (10)) and males from CL Apoyo (nA. zaliosus = 13 (11), nA. astorquii = 30 (9)), and a second one with females from CL Apoyo (nA. zaliosus = 25 (21), nA. astorquii = 36 (12)) and males from CL Xiloá (nA. sagittae = 22 (13), nA. amarillo = 36 (18)). The lack of replication for both experiments and the use of a limited number of families is a limitation of this experiment, which was discussed during the review process and is acknowledged in the publication.

Raw and intermediate data as well as code to reproduce results and figures are publicly available:
- Reference assembly can be accessed at NCBI: PRJNA643830
- Whole-genome resequencing data of all 453 samples in form of unmapped BAM files at ENA: PRJEB38173
- Transcriptomic data at NCBI SRA: PRJNA635556
- Geometric morphometric data, information on samples, and downstream data to reproduce our results can be downloaded from Dryad (10.5061/dryad.bcc2fqz91).
- Custom code used for the genome assembly (https://github.com/MartinPippel/DAmar) as well as custom code for genomic and morphometric analyses (https://github.com/alexnater/midas-genomics) can be accessed on GitHub.

Randomization

Within groups (i.e. lakes/species/morphs) individuals included in this study were sampled randomly. Groups were defined based on sampling location (lake), species characteristics, and morph (i.e. gold or dark; thin- or thick-lipped). As all subsequent analyses were performed on all individuals (for exceptions see Data exclusions above), no randomization was necessary. For mate choice experiments, the order of trials was randomized, but only one arena was available for the experiment.

Blinding

All measurements and morphometric data were collected blind to the identity of species and lake of origin of fish. Mate choice data was collected without knowledge of fish identities, as they were individually tagged with transponders, and only identified after they were taken out of any experiment (i.e. after pair formation and breeding).

Did the study involve field work? [x] Yes [ ] No

Field work, collection and transport

Field conditions	Fish for whole genome resequencing were collected over a twelve year time period. Environmental conditions were not relevant (i.e. for sampling) and are therefore not reported here.
Location	Adult fish (standard length =14.54 ± 3.43 cm (mean ± SD)) were collected in field expeditions of the Meyer lab to Nicaragua between 2003 and 2015. Fish were caught with gill nets or by harpooning in crater lakes Asososca León (12°26'08"N 86° 39'50"W), Asososca Managua (12°08'15"N 86°18'55"W), Apoyeque (12°14'38"N 86°20'31"W), Apoyo (11°55'23"N 86°01'57"W), Masaya (11°58'17"N 86°06'53"W), Tiscapa (12°08'22"N 86°16'15"W), and Xiloá (12°13'16"N 86°19'16"W) at water depths between 0 and 5m. Fish or tissue samples from the great lakes Nicaragua and Managua were obtained from local fishermen, mostly the big fish market in Granada (Lake Nicaragua fish; 11°56'00"N 85°57'20"W) and Mateares (Lake Managua fish; 12° 14'10"N 86°25'48"W).
Access and import/export	All field work and export of samples was approved by the local authorities, the Ministerio del Ambiente y los Recursos Naturales (MARENA), Nicaragua (permit numbers DGRNB-ACHL-0078, DGRNB-IC-006-2007, No. 026-11007/DGAP, DGPN/DB-27-2010, DGPN/DB/DAP-IC-0003-2012, DGPN/DB-02-2012, DGPN/DB-IC-004-2013, DGPN/DB-011-2014, DGPN/DB-IC-015-2015).
Disturbance	Midas cichlids are very abundant and a common food source in Nicaragua. The extractions of a limited number of animals for this study is therefore very unlikely to have caused any kind of disturbance of this natural system.

Reporting for specific materials, systems and methods

We require information from authors about some types of materials, experimental systems and methods used in many studies. Here, indicate whether each material, system or method listed is relevant to your study. If you are not sure if a list item applies to your research, read the appropriate section before selecting a response.

Materials & experimental systems

n/a	Involved in the study	
[x]	[ ]	Antibodies
[x]	[ ]	Eukaryotic cell lines
[x]	[ ]	Palaeontology
[ ]	[x]	Animals and other organisms
[x]	[ ]	Human research participants
[x]	[ ]	Clinical data

Methods

n/a	Involved in the study	
[x]	[ ]	ChIP-seq
[x]	[ ]	Flow cytometry
[x]	[ ]	MRI-based neuroimaging

Animals and other organisms

Policy information about studies involving animals; ARRIVE guidelines recommended for reporting animal research

Laboratory animals	Laboratory animals were bred and raised in the animal research facility of the University of Konstanz. We performed two different (sub)experiments. The first one with fish from the same lake and the second with fish from different lakes. Both of the experiments consisted of two trials (experimental groups) each. In the first trial, 114 adult fish (>2 years old) were available. A total of 48 of them were tested, comprising the limnetic species A. zaliosus (nfemales = 25 available (13 tested), nmales = 13 (12)) and the benthic species A. astorquii (nfemales = 30 (10), nmales = 46 (13)) from CL Apoyo. In the second trial, 115 (33 tested) adult fish were available, comprising the limnetic species A. sagittae (nfemales = 26 (6), nmales = 17 (6)) and the benthic species A. amarillo (nfemales = 12 (11), nmales = 60 (10)) from CL Xiloá. The trials were terminated when no more naïve fish of a certain sex and species were available for replacement (indicated as the final number of tested fish above). In the second (sub) experiment the first trial was conducted with females from CL Xiloá (nA. sagittae = 26 available (12 finally tested), nA. amarillo = 12 (10)) and males from CL Apoyo (nA. zaliosus = 13 (11), nA. astorquii = 30 (9)), and the second trial with females from CL Apoyo (nA. zaliosus = 25 (21), nA. astorquii = 36 (12)) and males from CL Xiloá (nA. sagittae = 22 (13), nA. amarillo = 36 (18)). All animals were kept at a constant 12:12 light-dark cycle and at a constant temperature of 28 degrees celsius. At no time were fish held on any kind of caloric restrictions.
Wild animals	This study did not involve experimentation on wild animals.
Field-collected samples	Laboratory strains of Midas cichlids were established prior to this study. Wild-caught animals were (as reported above under laboratory animals) also kept at a constant 12:12 light-dark cycle and at a constant temperature of 28 degrees celsius. At no time were fish held on any kind of caloric restrictions.
Ethics oversight	Euthanization of animals, animal husbandry, and mate choice experiments were approved by the German authorities (permit numbers T-16/13 and G-15/89, Regierungspräsidium Freiburg, Abteilung 3, Referat 35, Veterinärwesen & Lebensmittelüberwachung, Germany).

Note that full information on the approval of the study protocol must also be provided in the manuscript.
